# Antibacterial and anti-biofilm activities of Disaspidin BB against *Staphylococcus epidermidis*

**DOI:** 10.3389/fmicb.2023.999449

**Published:** 2023-01-19

**Authors:** Shihua Lan, Xiaofeng Chen, Chuanping Yin, Shengjun Xie, Shuaishuai Wang, Rongrong Deng, Zhibin Shen

**Affiliations:** ^1^School of Traditional Chinese Medicine, Guangdong Pharmaceutical University, Guangzhou, China; ^2^Guangzhou Hipower Pharmaceutical R&D Co., Ltd., Guangzhou, China; ^3^Guangdong Provincial Engineering Center of Topical Precise Drug Delivery System, Guangdong Pharmaceutical University, Guangzhou, China; ^4^Guangdong Cosmetics Engineering and Technology Research Center, Guangdong Pharmaceutical University, Guangzhou, China

**Keywords:** Disaspidin BB, antibacterial, *Staphylococcus epidermidis*, anti-biofilm activity, antibiotics resistance, anti-biofilm mechanism

## Abstract

**Introduction:**

*Staphylococcus epidermidis* infections are an important concern in worldwide, especially when associated with biofilms, and resistance of this agent to many drugs makes the situation even worse. We investigated the inhibitory effect of Disaspidin BB obtained from plant extracts and purifications on clinical *S. epidermidis* strains and their biofilms, and preliminarily investigated its mechanism of of its anti-biofilm activity.

**Methods and Results:**

The broth dilution method was used to determine the minimum inhibitory concentrations (MIC) of Disaspidin BB on 11 clinical *S. epidermidis* strains (MIC value of 0.63 ~ 2.5 μg/ml). SEP-05 was found to be erythromycin-resistant (MIC value>8 μg/ml) and Disaspidin BB sensitive with an MIC value of 0.63 μg/ml. The time-kill curve assay indicated that the antibacterial activity of Disaspidin BB against SEP-05 with concentration dependence. The metabolic activity and total biomass of the drug-treated SEP-05 biofilm in each stage were significantly inhibited by the crystalline violet and XTT assay, and the scavenging effect of Disaspidin BB on SEP-05 biofilm was also confirmed by SEM observation. The results of real-time quantitative PCR showed that subinhibitory concentrations Disaspidin BB can inhibit biofilm formation by affecting the expression level of key genes (*aap*, *atlE*, *icaA*, *luxS*, *recA*) in SEP-05 biofilm formation. In addition, the content of polysaccharides, proteins and extracellular DNA in biofilm matrix after the intervention of Disaspidin BB was significantly reduced, and it was tentatively determined that the ability of SEP-05 biofilm formation and its stability were thus disturbed.

**Discussion:**

The results show that Disaspidin BB has promising antibacterial effect on erythromycin-resistant *S. epidermidis* and significant scavenging effect on its biofilm, which provides a theoretical basis for the further development of BB as a new drug for the treatment of skin infections caused by *S. epidermidis*.

## 1. Introduction

*Staphylococcus epidermidis* is one of the most abundant bacterial colonizers in healthy human skin, but colonization by specific strains of *S. epidermidis* can be pathogenic in specific cases, harming the skin barrier (Brown and Horswill, 2020). Skin and soft tissue infections (SSTIs) are a common reason for patients seeking inpatient and outpatient medical care with more than 14 million out-patient visits a year (Burnham and Kollef, 2018), and *S. epidermidis* is one of the main pathogens related to SSTI (Rossolini and Stefani, 2009). *S. epidermidis* is able to colonize the human body or implanted medical devices and forming biofilms (Otto, 2014; Foster, 2020), the formation of which allows *S. epidermidis* to evade the host immune system (Kleinschmidt et al., 2015). Colonization of percutaneous devices or implanted medical devices allows bacteria access to the bloodstream and cause primary bacteremia, which is linked to the natural niche of *S. epidermidis* on human skin and ability to attach and form biofilm on foreign bodies (Rupp, 2014). In addition, Nosocomial bloodstream infections caused by CoNS are also strongly associated with *S. epidermidis* biofilm formation (Argemi et al., 2019), which can also lead to expansion and delayed healing of the infection (Reiter et al., 2013).

The biofilm state of bacteria is different from the planktonic state, which is a special form of existence adopted by bacteria to adapt to the external environment. Bacterial biofilm development basically consists of four successive stages: (1) attachment, (2) aggregation, (3) maturation, and (4) dispersion. During the attachment stage, planktonic cells adhere to biotic or abiotic surfaces and proliferate into sticky aggregations called microcolonies (also known as towers or mushroom-like structures). As these microcolonies develop, bacterial cells produce an extra cellular matrix that serves as a scaffold essential for establishing this three-dimensional architecture. Upon reaching a specific cell density, a mechanism is triggered to initiate extra cellular matrix degradation that releases cells embedded within the biofilm to disperse and reinitiate biofilm development at distal sites (Moormeier and Bayles, 2017). After forming the biofilm, bacteria can greatly resist the action of the body’s immune system and antibacterial drugs, and drug resistance is enhanced, rendering the traditional antibacterial strategies ineffective (Metcalf and Bowler, 2013). Studies have found that even high concentrations of vancomycin were unable to eliminate the biofilms of *S. aureus* and *S. epidermidis*, MICs were 2-, 4-, 8-, and up to 16-fold higher for biofilm cells than for planktonic cells (de Oliveira et al., 2016). Therefore, pharmacological treatment of biofilm-associated infections can be very challenging (Le et al., 2018).

The presence of the extracellular polymeric substance (EPS) is one of the characteristics of biofilms as microbial communities that contribute to the virulence of *staphylococcal* by forming a matrix that surrounds the cells, protects them from being killed by antimicrobial agents and host defenses, and helps them to attach to host tissues and abiotic surfaces (Kaplan et al., 2018). The components of EPS mainly include extracellular polysaccharides, proteins and eDNA (Arciola et al., 2015). The extracellular mixture of sugar polymers called extracellular polysaccharides is characteristic and critical for biofilm formation (Vuong et al., 2004). Extracellular proteins of biofilm mainly include functional metabolic and structural proteins, the former are proteases related to extracellular metabolism, the latter can mediate the adhesion process of the bacterium and specific binding to the organism, and can combine with extracellular proteins, together forming a complete system of biological periplasm (da Silva and De Martinis, 2013). Extracellular DNA, an important component of the biofilm matrix, is DNA secreted by bacteria on the outside of the bacterium after programmed death, and can facilitate the adhesion of the bacterium to the surface of biotic and abiotic materials through electrostatic adsorption during the biofilm adhesion stage (Wu and Xi, 2009). The role of eDNA is critical in the transition from attachment to accumulation during the early stages of *staphylococcal* biofilm formation (Whitchurch et al., 2002; Moormeier et al., 2014).

The quorum sensing system is an intercellular signaling mechanism that regulates gene expression and biological behavior by sensing changes in extracellular chemistry and concentration to exchange information between microorganisms (Singh and Ray, 2014). Biofilm is the form in which bacteria exist as a community; the presence of a quorum sensing system allows bacteria to communicate with each other through signals that control the formation of biofilms to adapt to environmental stresses. The expression of *aap*, *atlE*, *icaA*, *luxS*, and *recA* genes is closely related to the formation of *S. epidermidis* biofilm and are key genes related to extracellular proteins, autolysins, extracellular polysaccharides, quorum sensing, and stress response, respectively, associated with biofilm formation. During the adhesion period of the biofilm, the *atlE* protein transcribed by the *atlE* gene promotes bacteriophage autolysis, which leads to the secretion of extracellular DNA and thus mediates the production of bacteriophage adhesion (Zoll et al., 2010). During the period of *ica*-mediated aggregation, PIA, which is encoded by the *icaADBC* gene, promotes the formation of biofilm by adherent aggregation between colonies and can help *S. epidermidis* evade the immune response of the organism (Jeng et al., 2008). During the period of non-*ica* gene mediated aggregation, the *aap* protein transcribed by the *aap* gene can also promote colony formation, adhesion and aggregation to form biofilms (Yarawsky et al., 2017).

In Chinese folk medicine, *Dryopteris fragrans (L.) Schott* is used to treat various skin diseases such as psoriasis, rashes, dermatitis and acne. It has been found that the main active compounds of *D. fragrans* have a significant effect on a variety of skin diseases caused by fungi (Fan et al., 2012; Huang et al., 2016; Liu et al., 2018). Modern pharmacology has shown that phloroglucinols have antibacterial, antitumor, antioxidant, anti-inflammatory and antiviral activities, and nowadays there is an increasing interest in its antibacterial activity (Khan et al., 2020; Huang et al., 2021; Ismail et al., 2021). It was shown that the phloroglucinols such as aspidin BB, aspidin PB, aspidin AB, and pseudoaspidinol extracted from *D. fragrans* had significant antibacterial activity or antifungal activity against *Propionibacterium acnes*, *Staphylococcus aureus*, *S. epidermidis*, dermatophytes such as *Trichophyton rubrum*, *Trichophyton mentagrophytes* and *Microsporum canis* (Gao et al., 2016; Teng et al., 2018; Xin et al., 2018; Ye et al., 2018; Lin et al., 2019).

**Figure 1:**
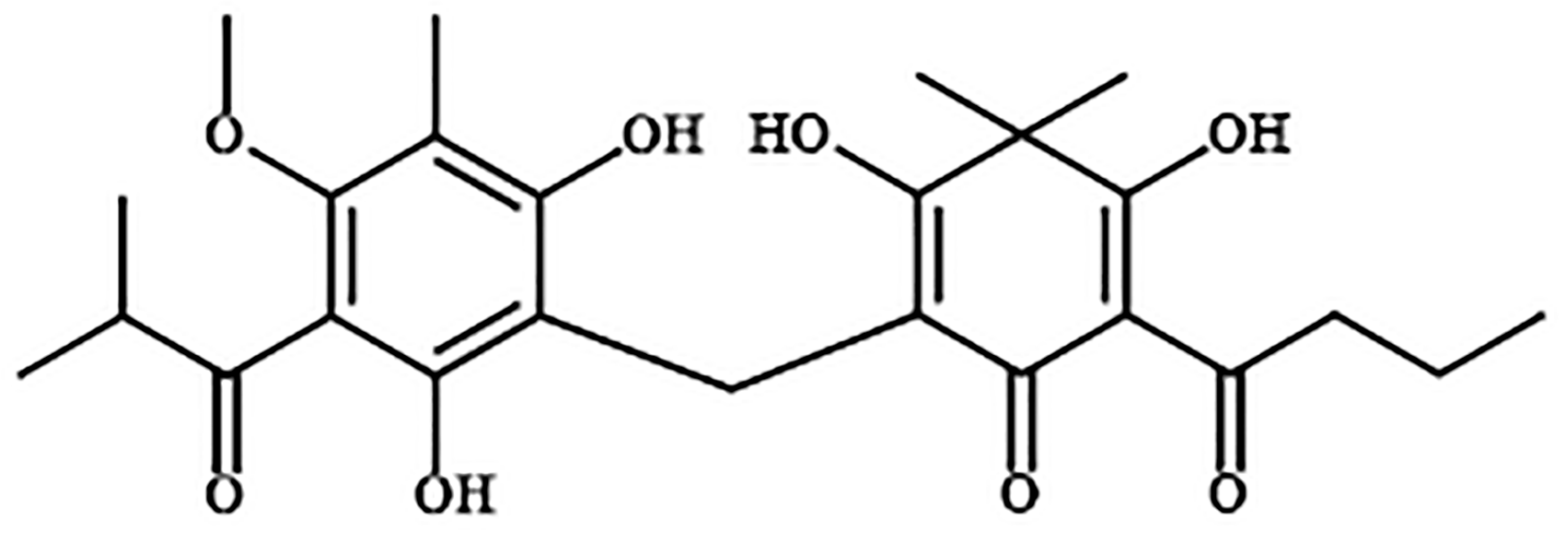
Structures of Disaspidin BB (Zheng et al., 2022).

Our group has isolated and extracted a new pseudoaspidinol compound Disaspidin BB from 50% ethanol extract of *D. fragrans*, and the structure is shown in Figure 1. Compared with the commonly used topical agents erythromycin or mupirocin, Disaspidin BB showed significant antibacterial activity against *S. epidermidis*, *Staphylococcus haemolyticus* and *methicillin-resistant S. aureus* (MRSA) all showed significant antibacterial activity, with the most sensitive to *S. epidermidis* with MIC values of 1.67–2.71 μg/ml (Zheng et al., 2022). Although erythromycin has good antibacterial activity, it has poor permeability and is prone to cross-or multi-drug resistance, and thus should not be the drug of choice. Although antibiotics can easily kill planktonic bacteria, they are often ineffective against biofilms, so the incomplete inhibition produced is likely to lead to drug-resistant reinfection. There are no studies on the effect of Disaspidin BB on the biofilm of *S. epidermidis*. Therefore, the aim of this study was to investigate the effect of Disaspidin BB on clinically isolated strains of *S. epidermidis* and their biofilms, as well as to preliminarily investigate the mechanism of its action on biofilms.

## 2. Materials and methods

### 2.1. Antimicrobial agents and chemicals

Disaspidin BB was made in the laboratory with a purity of 98%. Erythromycin (N0825A, > 97%) was purchased from Dalian Meilun Biotechnology Co., Ltd. Dimethyl sulfoxide (DMSO, Beijing Dingguo Changsheng biotechnology Co., Ltd) was used to prepare a stock solution of Disaspidin BB (16 mg/ml) and erythromycin (256 mg/ml), respectively, stored at 4°C in the dark.

### 2.2. Bacterial strain and growth conditions

Eleven clinical *S. epidermidis* strains (SE01-SE11), which were donated by Guangdong Lewwin Pharmaceutical Research Institute Co., Ltd. The quality control strain *Staphylococcus aureus* (ATCC 29213), was provided by Microbial Culture Collection Center of Guangdong (Guangdong, China). Bacteria were precultured on nutrient agar (NA) medium at 37°C for 24 h, and incubated in Caton-adjusted Mueller-Hnton broth (CAMHB) or tryptone soy broth (TSB) medium (Sigma Aldrich, United States) and then adjusted to 1 × 10^6^ CFU/ml using 0.5 McFarland standard.

### 2.3. Antibacterial susceptibility testing of Disaspidin BB on *Staphylococcus epidermidis*

#### 2.3.1. Minimum inhibitory concentration and minimum bactericidal concentration

The MIC and MBC assays were performed by the microdilution method according to the Clinical and Laboratory Standards Institute (Parvekar et al., 2020). In short, Add 1 × 10^6^ CFU/ml of the bacterial strain to individual wells of a microtiter plate after double dilution of test agent (160–0.31 μg/ml of Disaspidin BB and 2,560–5 μg/ml of erythromycin) with CAMHB medium. Plates was incubated at 37°C for 24 h, and the lowest concentration of the agent that inhibits the growth of *S. epidermidis* is considered the minimum inhibitory concentrations (MIC). The minimum bactericidal concentration (MBC) value was determined by adding 20 μl of the suspensions without bacterial turbidity on the surface of agar, and the lowest concentration without colony formation was MBC. *S.aureus* (ATCC 29213) was taken as the quality control strain and cefazolin was applied as the quality control drug. The assay was considered valid and reliable if the MIC of the QC strain was within the range of 0.25 to 1 μg/ml under the conditions of parallel operation.

#### 2.3.2. Time-kill curves

The time-kill curves test was performed based on the method provided by Barbour et al., (2014), with modifications. The strains were co-cultured with different concentrations of Disaspidin BB and erythromycin (1 × MIC, 2 × MIC, and 4 × MIC) at 37°C. Aliquots were collected at specified time points (0, 1, 2, 4, 6, 8, 12, and 24 h) and cell counts were performed as above. The rate of killing was determined by plotting CFU/mL against time to plot the time-kill curve.

### 2.4. Determination the inhibitory effect of Disaspidin BB on biofilms

#### 2.4.1. Determination of biofilm total biomass

Crystal violet staining test was used to detect the quantity of biomass of biofilm by Parai et al., (2020), with slight modification. Biofilms of adherent, aggregated and mature stages were formed by incubating SEP-05 in suspension at a concentration of 1 × 10^6^ CFU/ml in 96-well plates for 6, 18, and 36 h, respectively. After washing the wells to remove non-adherent cells, different concentrations of drugs (1 × MIC, 2 × MIC, and 4 × MIC Disaspidin BB and erythromycin) were added and continued to incubate for 24 h. Untreated samples were used as controls. Plates were washed 3 times with PBS and then 200 μl of methanol was added to anchor the biofilm for 30 min and discarded. Biofilms were stained with 200 μl of 0.1% crystal violet for 15 min and rinsed with PBS to remove unattached dye. Finally, the dye was dissolved with 95% ethanol and the biofilm biomass (OD_570_) was measured using a microplate reader (BIO-RAD, United States).

#### 2.4.2. Metabolic activity of biofilm assay

XTT testing was used to determine the metabolic activity of biofilm by Dong et al., (2020). The preliminary procedure was the same as the crystal violet assay. After coincubation with different concentrations of the drug for 24 h, the wells were washed and dried naturally, and 100 μl of XTT (0.5 mg/ml)/Vit.K3 (10 mM) reagent was added to each well. After incubation for 2 h at 37°C in the dark, OD_450_ was measured using a microplate reader (BIO-RAD, United States).

#### 2.4.3. Scanning electron microscope

According to a previous study, the SEM was used to observe the *S. epidermidis* cell morphological changes, according to the previous research with modification (Frassinetti et al., 2020). Sterile 14 mm circular slides were placed on 24-well plates and 1 ml of 1 × 10^6^ CFU/ml suspension of the test strain was added to each well and incubated at 37°C for 6, 18 and 36 h. Samples were dehydrated in an ascending ethanol series. The samples were sprayed with gold using a vacuum Ion sputtering instrument. Images were obtained using a SEM (JSM-7610FPlus, JEOL, Japan).

### 2.5. Determination of Disaspidin BB on biofilm matrix

#### 2.5.1. Preparation of drug-treated biofilm samples

The SEP-05 in suspension were co-cultured with different concentrations of Disaspidin BB (1 × MIC，1/2 × MIC, 1/4 × MIC and 1/8 × MIC) in 24-well plates at 37°C for 48 h, growth control group with TSB and SEP bacterial suspension. The formed biofilm was sonicated with sterile saline to obtain a suspension for centrifugation.

#### 2.5.2. Determination of extracellular polysaccharides in biofilm

Phenol-sulfuric acid method was used to determine the polysaccharides of biofilm. Glucose was used to draw the glucose standard curve by preparing a gradient series of concentrations of glucose solution and concentrated sulfuric acid and 6% phenol solution respectively, and then measuring the OD_490_ value, using the OD value as the vertical coordinate and the glucose concentration (μg/mL) as the horizontal coordinate, drawing the standard curve and plotting the linear regression equation. The biofilm supernatant was taken and reacted with concentrated sulfuric acid/phenol reagent to determine OD_490_, and the corresponding polysaccharide content was calculated on the glucose standard curve according to the OD value.

#### 2.5.3. Determination of extracellular protein in biofilm

The biofilm protein content was determined by BAC method. Extraction of extracellular protein from biofilms: the biofilm supernatant prepared in 2.5.1 was taken and sonicated 5 times, each time for 5 s with a gap of 5 s. After sonication, the samples were adjusted with sterile saline to OD_600 nm_ = 0.4, and 1 ml was taken into a sterile centrifuge tube and centrifuged at 4,000 r/min for 20 min at 4°C. The supernatant was then filtered through a 0.22 μm diameter membrane and placed in a new centrifuge tube.

The supernatant after centrifugation was measured using a total protein quantification kit (Jiancheng Biotech, Nanjing, China) and the total protein content of the sample was calculated using the following formula


$$\begin{array}{l} \mathrm{Total protein concentration}\left(\mu g/\mathrm{mL}\right)=\frac{OD_{562nm\;\mathrm{of sample}}-OD_{562nm\;\mathrm{of blank}}\;}{OD_{562nm\;\mathrm{of standard}}-OD_{562nm\;\mathrm{of blank}}\;}\;\times \;\;\mathrm{Concentration of standard}\left(524\;\mu g/\mathrm{mL}\right)\times \;\mathrm{Sample}\\ \mathrm{dilution multiple} \end{array}$$

#### 2.5.4. Determination of eDNA in biofilm

Nano Drop one C was used to determine the eDNA content of biofilm. The supernatant of the biofilm prepared in 2.5.1 was taken to add 100 μl of 0.5 mol/l EDTA pre-chilled at 4°C for 1 h for each sample, then discarded the liquid, rinsed with PBS 3 times. The biofilm was resuspended with 50 mmol/l TEN buffer, adjusted to OD_600nm_ = 0.4, and 1 ml was transferred to a pre-cooled centrifuge tube and centrifuged at 18,000 r/min for 5 min at 4°C. The supernatant was extracted with a bacterial genomic DNA extraction kit (Solarbio, Beijing, China) to obtain the eDNA-containing solution. The eDNA concentration values of the solutions containing eDNA were determined with Nano Drop one C.

### 2.6. Expression of genes related to biofilm formation and quorum sensing

#### 2.6.1. RNA extraction and cDNA synthesis and qRT-PCR

The *aap*, *atlE*, *icaA*, and *luxS* genes expression during the biofilm formation were quantified by measuring the transcript levels of the genes *via* qRT-PCR. The gene-specific primers were synthesized by Shanghai Sangon Biotechnology Co., Ltd. (Table 1). Biofilms with different concentrations of Disaspidin BB (1/8 × MIC, 1/4 × MIC, 1/2 × MIC, 1 × MIC) were harvested, centrifuged, resuspended in Trizol reagent (Invitrogen, United States) with the manufacturer’s instructions to extract mRNA and transferred to RNase-free 1.5 ml microcentrifuge tubes.

**Table 1:** Primer sequences of target genes.

Genes	Primer sequence (5′–3′)	Product Length (bp)
16SrRNA-F	GGCAAGCGTTATCCGGAATT	101
16SrRNA-R	GTTTCCAATGACCCTCCACG	
Aap-F	TGTCCCATACCCTCTATAGCCTTG	104
Aap-R	CACCTAGTGCAGCTGGTTTCAG	
AtlE-F	ACAAATGCGTGTACGAATGCA	112
AtlE-R	GACGTCCTGAAGGTATCGTTGTT	
IcaA-F	AGTACGAACCACGTGCTCTATGC	100
IcaA-R	AGTACTTCATGCCCGCCTTG	
LuxS-F	CCGGGACTATGGAAGGTCTTAA	103
LuxS-R	AAGGAATGTAGACCAGGCATATCC	
RecA-F	ATGCCGAACATGCTCTCGAT	93
RecA-R	CTTGTTCACCATGATCAGGTTGAG	

The total RNA was extracted by TRIzol method as follows: take an appropriate amount of biofilm precipitate in a centrifuge tube and add 1 ml Trizol, vibrated to make uniform and leaved at room temperature for 5 min, 0.2 ml of chloroform was added, shaken with a shaker for 15 s, and then incubated for 3 min at room temperature, centrifuged at 12000 r/min for 15 min at 4°C, and the supernatant was taken to a new 1.5 ml. An equal volume of isopropanol was added to the supernatant, incubated the sample at –20°C for 30 min, then centrifuged at 12000 r/min at 4°C for 10 min, discarded the supernatant. The precipitate was washed with 800 μl 75% ethanol (containing DEPC water), centrifuged at 7500 r/min for 5 min at 4°C, and discarded the ethanol; dried under vacuum for 10 min, added 20–50 μl of DEPC-treated water to dissolve the RNA, stored at-80°C for backup.

Purified RNA was dissolved in 20 ml of DEPC-treated water and set aside. Four microliter of RNA sample was taken as template and cDNA was synthesized by reverse transcription reaction using the Prime Script RT kit (Takara, Japan) following the manufacturer’s protocol.

The qPCR reactions were performed by SYBR Premix EX Taq II kit (Takara, Japan) following the manufacturer’s instructions. The qPCR reaction mixture (20 ml) contained 2 μl of cDNA, 10 μl of SYBR Premix Dimer Eraser, 0.6 μl of 10 μM of each primer, and 6.8 μl of sterile double RNase treated water. qPCR conditions included initial denaturation at 95°C for 3 min, followed by 40 cycles of amplification at 95°C for 5 s denaturation, annealing at 60°C for 30 s, and extension at 72°C for 30 s. The relative expression of genes was calculated by the 2^-ΔΔCt^ method, normalized using the 16S rRNA gene of *S. epidermidis* as an internal standard.

### 2.7. Statistical analysis

All assays were performed in triplicate. Analyses were performed using GraphPad Prism software version 8.0 (GraphPad Software, Inc., La Jolla, CA). The differences were evaluated with a one-way ANOVA. The differences were considered significant when *p* < 0.05.

## 3. Results

### 3.1. The result of antibacterial susceptibility testing of Disaspidin BB on *Staphylococcus epidermidis*

#### 3.1.1. The MIC and MBC

Table 2 shows that the MIC of Disaspidin BB against 11 strains of *S. epidermidis* ranged from 0.63 to 2.5 μg/ml, and MBC ranges from 0.94 to 26.67 μg/ml. Among them, SEP-03 ~ 08 and SEP-11 were all resistant to erythromycin (MIC >8 μg/ml) according to the document M100-S22 issued by CLSI (Ceriotti et al., 2012). In contrast, Disaspidin BB showed significant bacterial inhibition against these clinically resistant strains of *S. epidermidis*. Therefore, we selected SEP-05 strain, which is both erythromycin-resistant and Disaspidin BB-susceptible (MIC value of 0.63 μg/ml), as the test strain for the follow-up experiment. Meanwhile, the MIC of quality control strain *S. aureus* 29,213 to benzathine was 0.156 μg/ml, which was within the range of 0.12–0.5 μg/ml, indicating that the results of this experiment were reliable.

**Table 2:** The MIC and MBC results of Disaspidin BB on *Staphylococcus epidermidis.*

Strains	Drugs(μg/mL)			
	Disaspidin BB		Erythromycin	
	MIC	MBC	MIC	MBC
SEP-01	1.67 ± 0.59**	9.17 ± 7.73	0.26 ± 0.07	1.67 ± 0.59
SEP-02	0.63 ± 0.00**	1.04 ± 0.29	0.13 ± 0.04	0.73 ± 0.39
SEP-03	1.04 ± 0.29**	4.58 ± 3.86	73.33 ± 14.90	120.00 ± 40.00
SEP-04	2.50 ± 0.00**	26.67 ± 9.43	120.00 ± 40.00	906.67 ± 527.97
SEP-05	0.63 ± 0.00**	20.00 ± 0.00	160.00 ± 0.00	373.33 ± 199.56
SEP-06	1.04 ± 0.29**	26.67 ± 9.43	160.00 ± 0.00	533.33 ± 150.85
SEP-07	1.25 ± 0.00**	5.00 ± 0.00	133.33 ± 37.71	>2560.00
SEP-08	0.63 ± 0.00**	1.67 ± 0.59	53.33 ± 18.86	106.67 ± 37.71
SEP-09	0.73 ± 0.23**	1.25 ± 0.00	0.16 ± 0.00	3.75 ± 1.25
SEP-10	0.63 ± 0.00**	3.75 ± 1.77	0.31 ± 0.00	1.46 ± 0.78
SEP-11	0.73 ± 0.23**	0.94 ± 0.31	10.00 ± 0.00	33.33 ± 9.43

SEP-01 ~ 11 means clinical strains of *S. epidermidis* 01 ~ 11; **means *p* < 0.01.

#### 3.1.2. Time-kill curves

**Figure 2:**
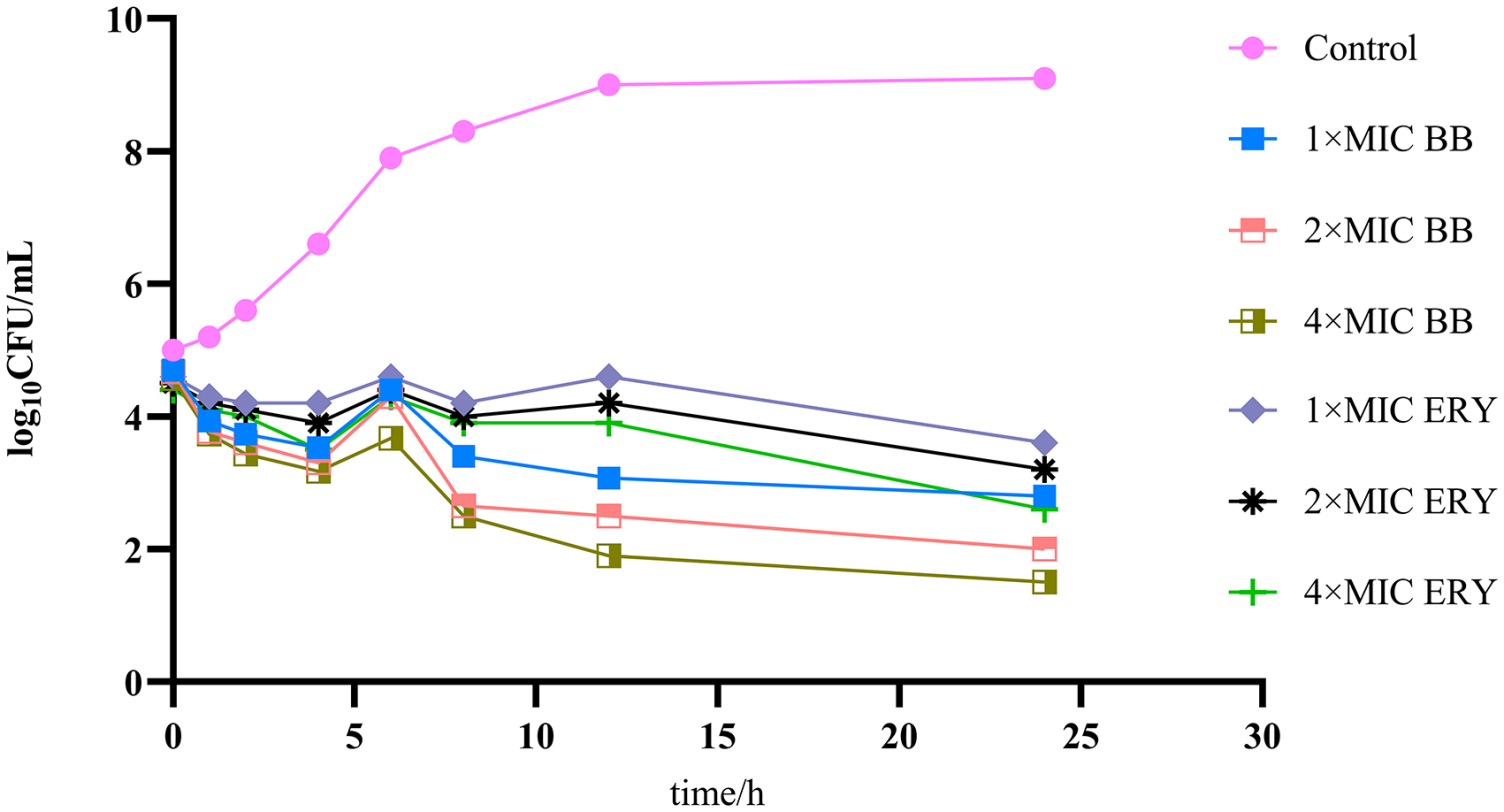
The time-kill curves of drugs on SEP-05. BB, Disaspidin BB. ERY, erythromycin.

The results of the time-kill curves of Disaspidin BB and erythromycin on SEP-05 are shown in Figure 2. From the results, it can be seen that SEP-05 entered the logarithmic growth period at 2-12 h and the stable growth period at 12-24 h. In the Disaspidin BB administration group, Disaspidin BB with 1 × MIC, 2 × MIC, and 4 × MIC decreased SEP-05 by 1.87, 2, and 2.17 log_10_ CFU/mL at 2 h, 5.93, 6.5, and 7.1 log_10_ CFU/mL at 12 h, 6.3, 7.1, and 7.6 log_10_ CFU/mL at 24 h, respectively. In the erythromycin administration group, erythromycin with 1 × MIC, 2 × MIC, and 4 × MIC decreased SEP-05 by 1.4, 1.5, and 1.6 log_10_ CFU/mL at 2 h, 4.4, 4.8, and 5.1 log_10_ CFU/mL at 12 h, 5.5, 5.9, and 6.5 log_10_ CFU/mL at 24 h, respectively.

Disaspidin BB showed bacteriostatic effect at 2 h (decreased by 1.87–2.17 log_10_ CFU/mL) and bactericidal effect at 12, 24 h (decreased by 5.93–7.6 log_10_ CFU/mL) (Skariyachan et al., 2020), and the effect was concentration-dependent. Disaspidin BB had a significant effect on SEP-05 throughout the growth period, and the effect of Disaspidin BB with 1 × MIC,2 × MIC,4 × MIC was better than that of erythromycin with 1 × MIC,2 × MIC,4 × MIC throughout the period of action.

### 3.2. The result of inhibitory effect of Disaspidin BB on SEP-05 biofilms

#### 3.2.1. Effects of Disaspidin BB and erythromycin on total biomass quantity of biofilm

**Figure 3:**
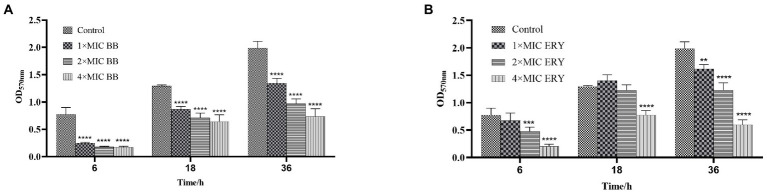
The quantity of biomass at each stage of biofilm formation of SEP-05. **(A)** SEP-05 treated with Disaspidin BB; **(B)** SEP-05 treated with erythromycin. *means *p* < 0.05, **means *p* < 0.01, ***means *p* < 0.001, ****means *p* < 0.0001.

The results of total biofilm after treatment with different concentrations of Disaspidin BB (Figure 3A) and erythromycin (Figure 3B) on SEP-05 biofilm in the adherence, aggregation and maturation phases (6 h, 18 h, 24 h) are shown in Figure 3.

In the adherence stage (6 h), 1 × MIC, 2 × MIC, and 4 × MIC of Disaspidin BB reduced the total biomass by 67.63, 76.86, and 77.54% (*p* < 0.0001), respectively. 1 × MIC of Erythromycin had little effect, while 2 × MIC and 4 × MIC of erythromycin reduced the total biomass by 38.56 and 73.46% (*p* < 0.001), respectively.

In the aggregation stage (18 h), Disaspidin BB at 1 × MIC, 2 × MIC, and 4 × MIC reduced the total biomass by 32.62, 44.89, and 50.19% (*p* < 0.0001), respectively. Erythromycin at 1 × MIC and 2 × MIC had no effect, and erythromycin at 4 × MIC reduced the total biomass by 40.26% (*p* > 0.05).

At maturity stage (36 h), Disaspidin BB reduced the total biomass by 32.30, 51.25, and 62.97% for 1 × MIC, 2 × MIC, and 4 × MIC, respectively (*p* < 0.0001). Erythromycin reduced the total biomass by 18.61, 38.28, and 69.93% for 1 × MIC, 2 × MIC, and 4 × MIC, respectively (*p* < 0.01).

In conclusion, Disaspidin BB had a scavenging effect on all stages of biofilm, and the scavenging effect increased with the increase of concentration, and the effect of Disaspidin BB on the total biomass of all stages was better than that of the positive drug erythromycin.

#### 3.2.2. Effects of Disaspidin BB and erythromycin on metabolic activity of biofilm

**Figure 4:**
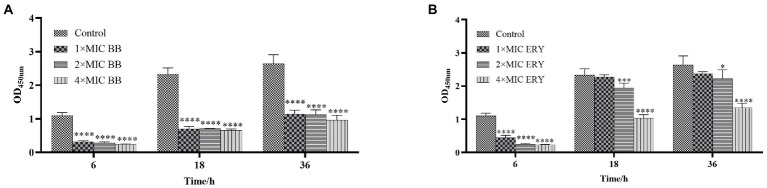
The metabolic activity at each stage of biofilm formation of SEP-05. **(A)** SEP-05 treated with Disaspidin BB; **(B)** SEP-05 treated with erythromycin. *means *p* < 0.05, **means *p* < 0.01, ***means *p* < 0.001, ****means *p* < 0.0001.

The results of biofilm metabolic activity after the action of different concentrations of Disaspidin BB (Figure 4A) and erythromycin (Figure 4B) on the biofilm of SEP-05 at the stages of adhesion, aggregation and maturation (6 h, 18 h, 24 h) were measured by XTT method as shown in Figure 4.

During the adhesion stage (6 h), Disaspidin BB at 1 × MIC, 2 × MIC, and 4 × MIC reduced the metabolic activity of the biofilm by 71.35, 74.11, and 78.02%, respectively (*p* < 0.01). Erythromycin at 1 × MIC, 2 × MIC, and 4 × MIC reduced the metabolic activity of the biofilm by 59.91, 77.52, and 79.16% (*p* < 0.01).

During the aggregation stage (18 h), Disaspidin BB at 1 × MIC, 2 × MIC, and 4 × MIC reduced the metabolic activity of the biofilm by 69.96, 69.89, and 71.74%, respectively (*p* < 0.01). Erythromycin at 1 × MIC had no effect, and erythromycin at 2 × MIC and 4 × MIC reduced the metabolic activity of the biofilm by 17.07, and 55.79% (*p* < 0.01).

During the maturation stage (36 h), Disaspidin BB at 1 × MIC, 2 × MIC, and 4 × MIC reduced the metabolic activity of biofilm by 59.94, 57.36, and 63.60%, respectively (*p* < 0.01). Erythromycin at 1 × MIC had little effect, and erythromycin at 2 × MIC and 4 × MIC reduced the metabolic activity of biofilm by 15.92 and 48.99% (*p* < 0.05).

In conclusion, Disaspidin BB significantly reduced the metabolic activity of biofilm at all stages of biofilm with concentration dependence, and it can be seen that the effect of Disaspidin BB on the metabolic activity of biofilm at all stages of biofilm was better than that of the positive drug erythromycin.

#### 3.2.3. Scanning electron microscope analysis

**Figure 5:**
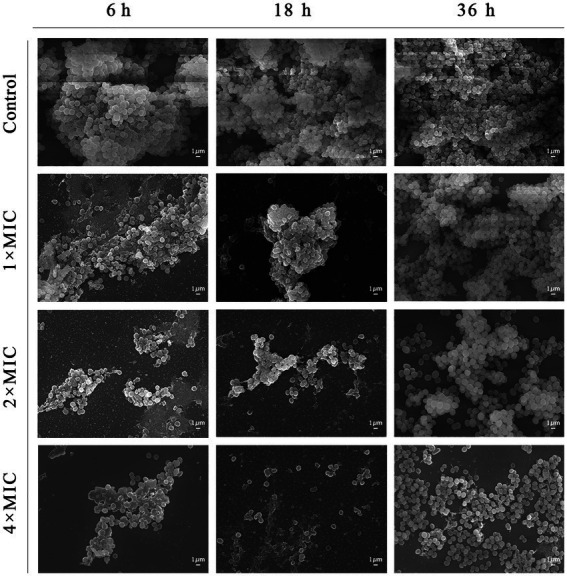
SEM images showing the effects of Disaspidin BB on SEP-05. Magnification: ×5,000.

**Figure 6:**
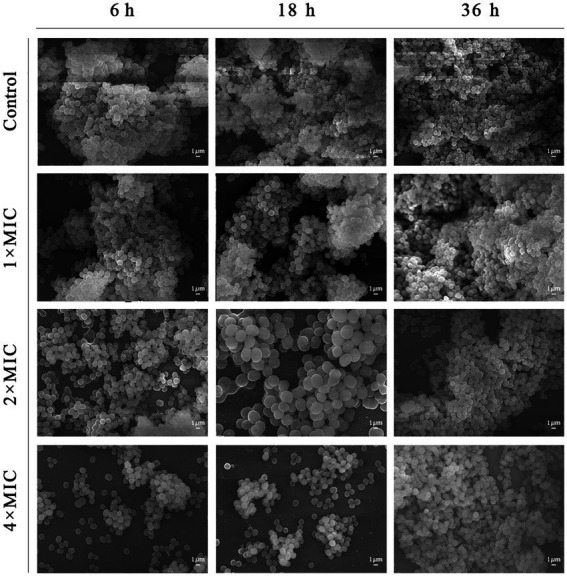
SEM images showing the effects of erythromycin on SEP-05. Magnification: ×5,000.

The results of microscopic morphological and structural changes of the SEP-05 biofilm at different stages after intervention with Disaspidin BB and erythromycin, respectively, were observed by SEM as shown in Figures 5, 6.

During the adhesion stage (6 h), the morphological structure of the biofilm was intact in the control group. The biofilm structure was significantly affected by the administration of Disaspidin BB, both the extracellular matrix and the amount of viable bacteria decreased with increasing drug concentration, and the bacterium structure in the 2 × MIC BB administration group showed depression and deformation. The bacterium in the 4 × MIC BB administration group ruptured.

In the aggregation stage (18 h), the biofilm morphology of the control group was intact, and a large amount of extracellular matrix was secreted and wrapped around the bacterium and aggregated into clusters with each other. The amount of extracellular matrix was reduced in the 1 × MIC Disaspidin BB administration group. 2 × MIC Disaspidin BB administration group tended to disintegrate the biofilm structure, and 4 × MIC Disaspidin BB administration group was structurally unformed, with only a few crumpled, deformed organisms present. The 1 × MIC erythromycin administration group basically had no effect on the structure of the biofilm, and the amount of biofilm in the 2 × MIC and 4 × MIC erythromycin administration groups decreased, but the biofilm structure still existed.

At the maturation stage (36 h), the control group had a complete morphological structure with a large amount of extracellular matrix wrapped around the bacterium, and the extracellular matrix was tightly connected, forming a dense three-dimensional structure. The number of extracellular matrix and bacterium in the 2 × MIC Disaspidin BB administration group was significantly reduced, but the biofilm structure still existed; the three-dimensional structure of biofilm in the 4 × MIC Disaspidin BB administration group tended to disintegrate and the bacteriophage appeared deformed. There was a slight decrease in the number of organisms within the biofilm in the 1 × MIC, 2 × MIC, and 4 × MIC erythromycin administration groups.

### 3.3. Effect of Disaspidin BB on the biofilm matrix of SEP-05

#### 3.3.1. Effect of Disaspidin BB on the biofilm extracellular polysaccharide of SEP-05

**Figure 7:**
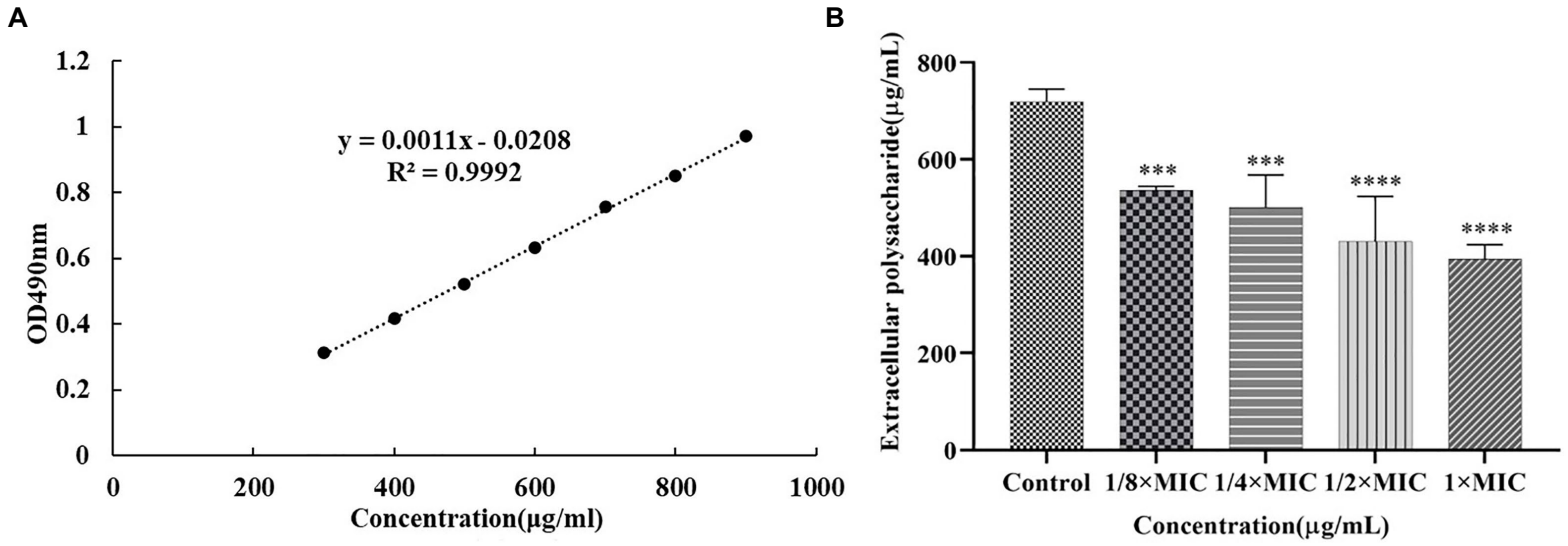
Effect of Disaspidin BB on the biofilm extracellular polysaccharide of SEP-05. **(A)** glucose standard curve. **(B)** Changes in the amount of biofilm extracellular polysaccharides of SEP-05 by Disaspidin BB administration. ***means *p* < 0.001. ****means p < 0.0001.

The effects of Disaspidin BB on the extracellular polysaccharide of SEP-05 biofilm were shown in Figure 7. 1/8 × MIC, 1/4 × MIC, 1/2 × MIC, and 1 × MIC of Disaspidin BB decreased the extracellular polysaccharide of biofilm by 25.42, 30.38, 40.12, and 45.21%, respectively. After different concentrations of Disaspidin BB treatment, the amount of extracellular polysaccharide in the biofilm decreased significantly (*p <* 0.001) compared with the growth control group, and the amount of extracellular polysaccharide decreased gradually with the increase of drug treatment concentration.

#### 3.3.2. Effect of Disaspidin BB on the biofilm extracellular protein of SEP-05

**Figure 8:**
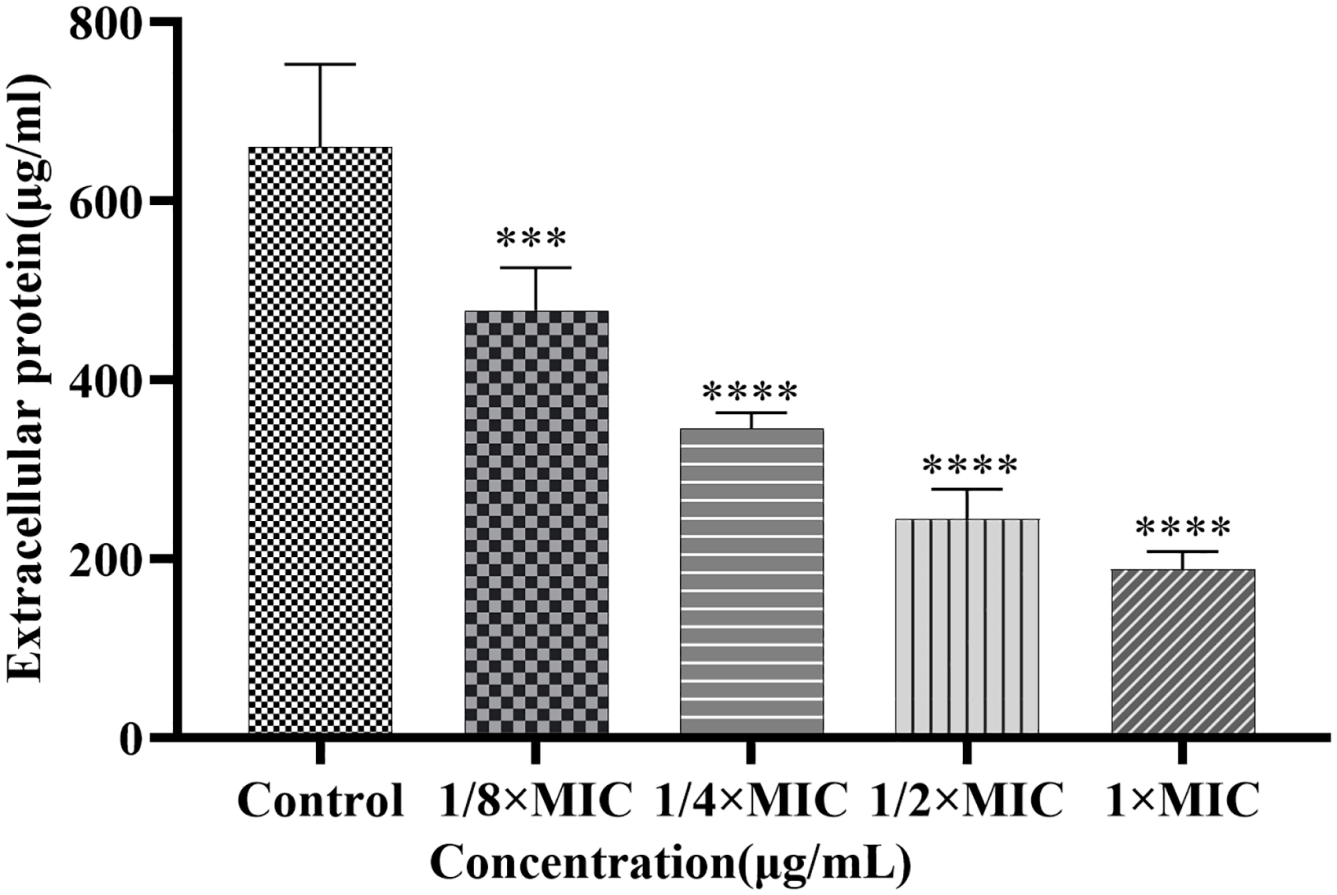
Effect of Disaspidin BB on the biofilm extracellular protein of SEP-05. ***means *p* < 0.001. ****means *p* < 0.0001.

The effects of Disaspidin BB on the extracellular protein of SEP-05 biofilm were shown in Figure 8. 1/8 × MIC, 1/4 × MIC, 1/2 × MIC, and 1 × MIC of Disaspidin BB decreased the extracellular protein of biofilm by 8.86, 31.65, 59.92, and 69.20%, respectively. After different concentrations of Disaspidin BB treatment, the amount of extracellular protein in the biofilm decreased significantly (*p <* 0.001) compared with the growth control group, and the amount of extracellular protein decreased gradually with the increase of drug treatment concentration.

#### 3.3.3. Effect of Disaspidin BB on the biofilm extracellular DNA of SEP-05

**Figure 9:**
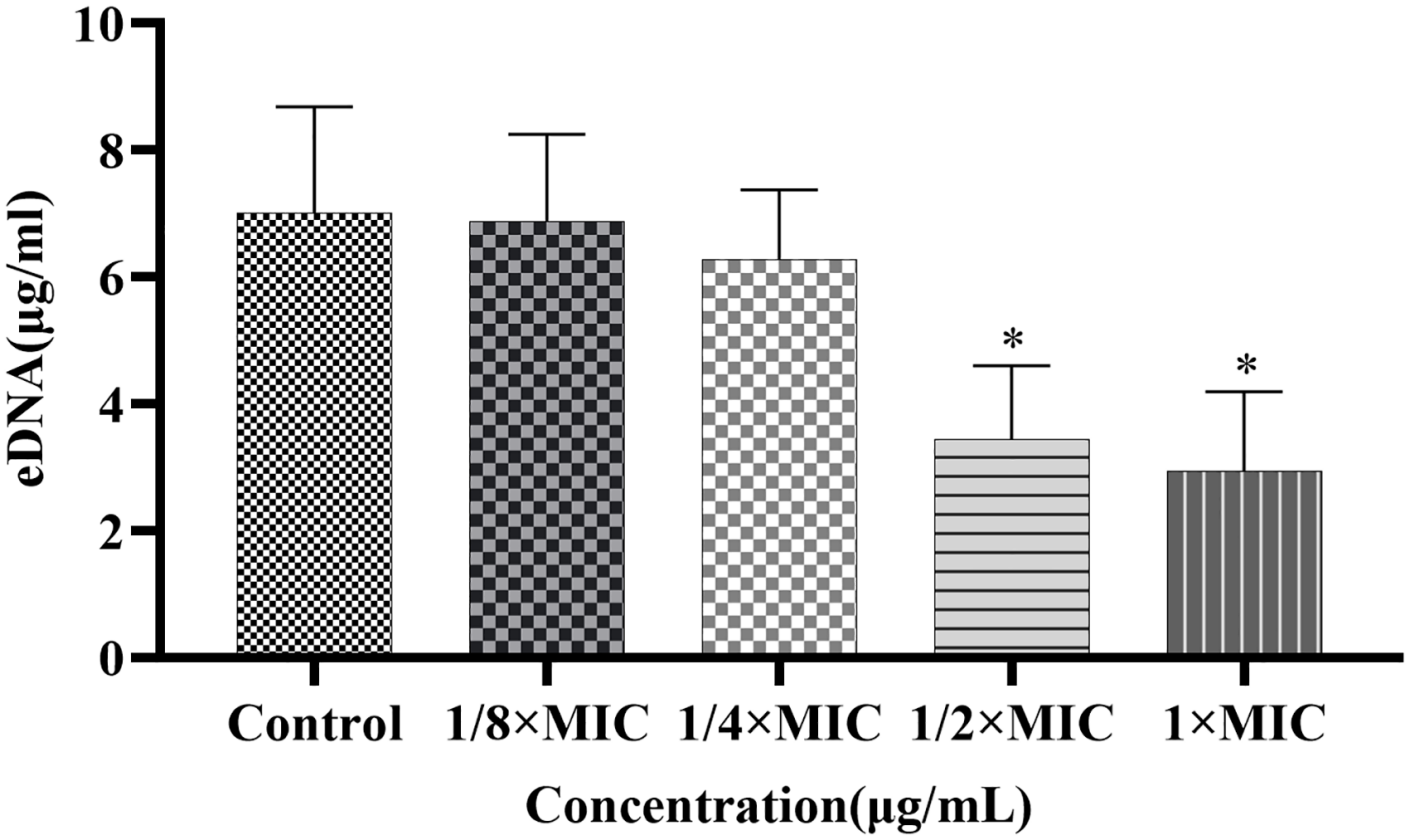
Effect of Disaspidin BB on the biofilm extracellular DNA of SEP-05. *means *p* < 0.05.

The effects of Disaspidin BB on the eDNA of SEP-05 biofilm were shown in Figure 9. 1/8 × MIC, 1/4 × MIC, 1/2 × MIC, and 1 × MIC of Disaspidin BB decreased the extracellular protein of biofilm by 1.90, 10.4, 50.95, and 58.1%, respectively. When the treatment concentration of Disaspidin BB reached 1/2 × MIC, the release of eDNA decreased significantly (*p <* 0.05), and the release of eDNA decreased gradually with the increase of drug treatment concentration.

In general, the biofilms formed after intervention with Disaspidin BB showed a significant reduction in the content of both extracellular polysaccharides and proteins. The inhibition of extracellular polysaccharides and proteins led to the disturbance of the adhesion and maturation process of the biofilm, and the ability of biofilm formation was greatly reduced.

### 3.4. Expression levels of genes of SEP-05 following Disaspidin BB

**Figure 10:**
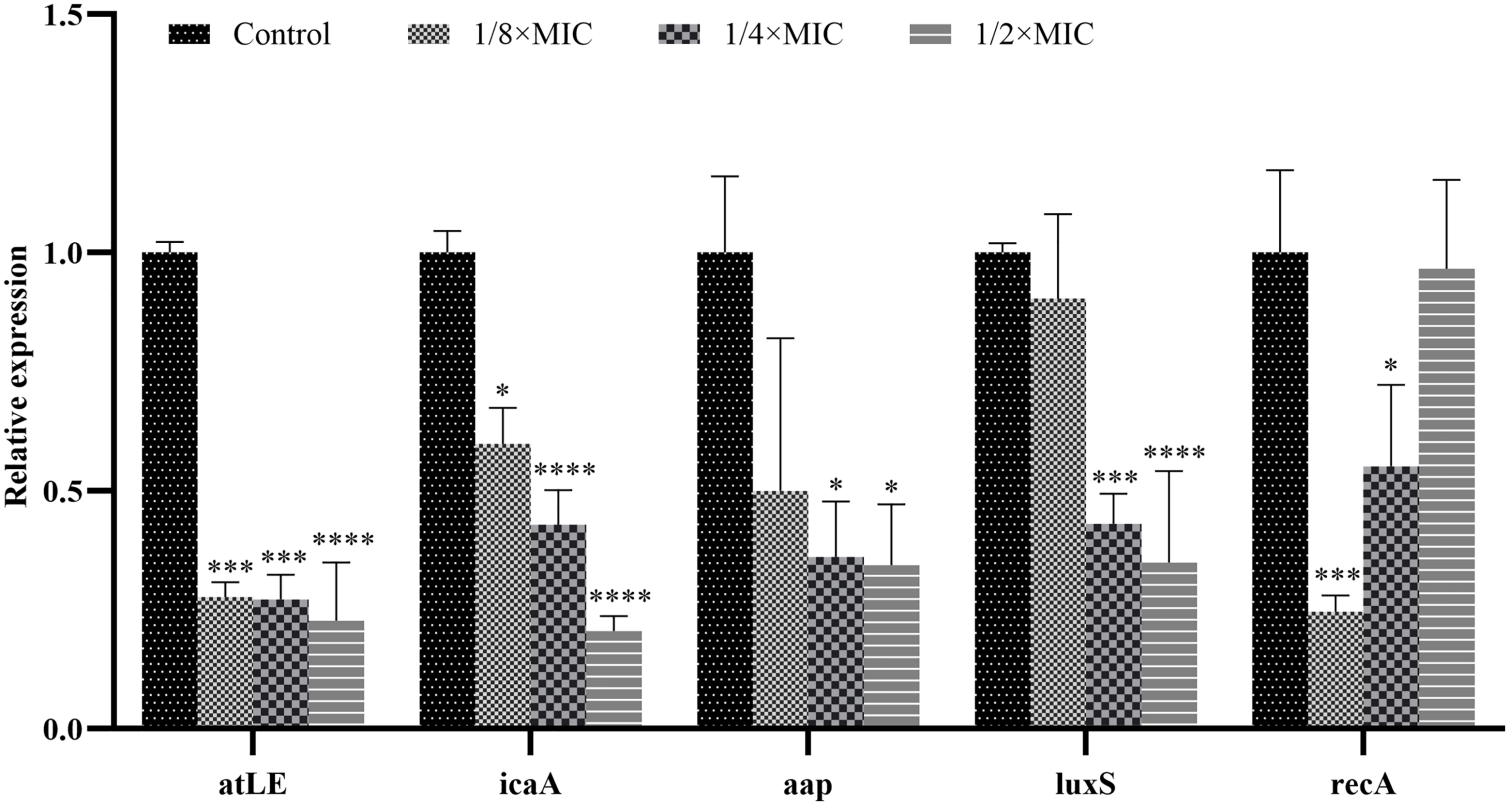
Expression of key genes in SEP-05 biofilm formation after Disaspidin BB intervention. * means *p* < 0.05, **means *p* < 0.01, ***means *p* < 0.001, ****means *p* < 0.0001.

From the results shown in Figure 10, it is clear that the expression of *aap*, *atlE*, *icaA*, and *luxS* genes can be reduced by Disaspidin BB intervention at subinhibitory concentrations.

For the *atlE* gene, the expression of *atlE* decreased by 72.40, 72.84, and 77.30% after treatment with 1/8 × MIC, 1/4 × MIC, and 1/2 × MIC of Disaspidin BB, respectively, relative to the growth control.

For the *icaA* gene, the expression of *icaA* decreased by 40.24, 57.17 and 79.50% after Disaspidin BB treatment with 1/8 × MIC, 1/4 × MIC, and 1/2 × MIC, respectively, relative to the growth control.

For the *aap* gene, the expression of *aap* decreased by 50.06, 63.96, and 65.65% after Disaspidin BB treatment with 1/8 × MIC, 1/4 × MIC, and 1/2 × MIC, respectively, relative to the growth control.

For *luxS* gene, the expression of *luxS* decreased by 9.65, 56.99, and 65.09% after treatment with 1/8 × MIC, 1/4 × MIC, and 1/2 × MIC of Disaspidin BB, respectively, relative to the growth control.

For the *recA* gene, *recA* expression decreased by 75.44, 44.91, and 3.45% after 1/8 × MIC, 1/4 × MIC, and 1/2 × MIC Disaspidin BB treatment, respectively, relative to the growth control.

Subinhibitory concentrations of Disaspidin BB significantly inhibited the expression of *icaA*, *atlE*, *aap*, and *luxS* genes (*p* < 0.05), and the inhibitory effect increased with increasing concentration. It is suggested that it can inhibit biofilm formation by affecting changes in the expression levels of key genes (*aap*, *atlE*, *icaA*, *luxS*, *recA*) in biofilm formation in *S. epidermidis*.

## 4. Discussion

We screened 8 erythromycin-resistant *S. epidermidis* strains (MIC>8 μg/ml) from 11 clinical strains of *S. epidermidis* by MIC value determination, and Disaspidin BB showed significant inhibition effect on all 11 strains of *S. epidermidis* with MIC value of 0.63 ~ 2.50 μg/ml. From a series of erythromycin-resistant strains, we selected the most Disaspidin BB-sensitive strain SEP-05 (MIC 0.63 μg/ml) for follow-up experiments. Time-kill curves assays are commonly used in laboratories to evaluate the dynamic antimicrobial activity of antimicrobial drugs, the result demonstrating that Disaspidin BB at 0.625–1.25 μg/ml could have a bacteriostatic or bactericidal effect on the whole growth cycle of *S. epidermidis*.

Current therapeutic approaches to infections associated with biofilms generally focus on the early and mature stages of biofilm formation. In the early stage of biofilm formation, the number of bacteria is small and the amount of extracellular matrix secretion is not much, so the barrier effect of antibacterial drugs is not strong, and the formation of biofilm can be effectively inhibited by inhibiting its adhesion through drug interference (Cheng et al., 2012). During the biofilm maturation stage, the structural integrity of the biofilm is destroyed by various means to kill the encapsulated bacterium, but at this time the resistance of the biofilm to various adverse factors is at its strongest, and it is difficult to effectively remove the bacterial biofilm (Lister and Horswill, 2014). The scavenging effect of Disaspidin BB on *S. epidermidis* biofilm at the stages of adhesion, aggregation and maturation was determined by crystal violet assays and XTT methods. The results showed that the highest biofilm scavenging rates of 0.63–1.25 μg/ml of Disaspidin BB were up to 77.54, 50.19, and 62.97% for *S. epidermidis* during the adherence, aggregation, and maturation stages of biofilm, respectively, while the concentration at which erythromycin achieved the same scavenging effect was 640 μg/ml, which was 80 times higher than the prescribed resistance concentration. The results of SEM further verified that Disaspidin BB could effectively disrupt the three-dimensional structure of the biofilm of *S. epidermidis* at all stages, reduce the number of bacteria within the biofilm and decrease the production of its extracellular matrix.

To further investigate the mechanism of action of Disaspidin BB on *S. epidermidis* biofilm, the effect of Disaspidin BB on polysaccharides, proteins and eDNA, the main components of biofilm matrix, was measured. The results showed that the biofilm formed after the intervention with subinhibitory concentrations of Disaspidin BB showed a significant decrease in the content of both extracellular polysaccharides and proteins. The inhibition of extracellular polysaccharides and proteins led to the disturbance of the adhesion and maturation process of the biofilm, and the ability of biofilm formation was greatly reduced. In this experiment, the eDNA content of *S. epidermidis* biofilm treated with subinhibitory concentrations of Disaspidin BB was significantly reduced, indicating that Disaspidin BB can affect the adhesion stage of biofilm formation and reduce the stability of mature biofilm.

Disaspidin BB inhibits biofilm formation by affecting the expression levels of key genes (*aap*, *atlE*, *icaA*, *luxS*, *recA*) in *S. epidermidis* biofilm formation. *LuxS* genes are the most widespread genes in the quorum sensing system. The synthesis of AI-2 catalyzed by the *luxS* system downregulates the expression of genes associated with biofilm formation, thereby inhibiting biofilm formation (Hardie and Heurlier, 2008). It was shown that knockdown of *LuxS* gene in *S. epidermidis* significantly upregulated the expression of *icaA*, *atlE*, and *aap* genes, which greatly promoted the biofilm formation, while the biofilm formation ability returned to the initial state after the addition of AI-2 and continued incubation (Xu et al., 2006). The results showed that subinhibitory concentrations of Disaspidin BB significantly inhibited the expression of *icaA*, *atlE*, *aap* and *luxS* genes, and the inhibitory effect increased with increasing concentration. It has been shown that the stress response (SOS response) occurs when bacteria are exposed to high environmental stress or severe DNA damage affecting transcriptional expression. *RecA* gene is a key gene in the bacterial stress response, which can be activated to repair damaged DNA in case of DNA damage and maintain the integrity of the genome (Podlesek and Žgur Bertok, 2020). Therefore, we speculate that an increase in the concentration of Disaspidin BB would reduce the downregulation of *recA* gene expression following the action of *S. epidermidis* for reasons related to the SOS response.

The results showed that Disaspidin BB significantly inhibited the planktonic bacteria and biofilm of clinical strains of *S. epidermidis*, and its mechanism of inhibition was tentatively related to the inhibition of biofilm matrix and the inhibition of gene expression related to biofilm formation and quorum sensing. This study provides a theoretical basis for the use of Disaspidin BB as a drug for the treatment of skin infections. However, this study only established the static biofilm *in vitro*, while the biofilm formation in the biological organism varies greatly due to the complex environment and many influencing factors, and the *in vivo* model of biofilm should be studied subsequently.

## Data availability statement

The original contributions presented in the study are included in the article/supplementary material, further inquiries can be directed to the corresponding authors.

## Author contributions

SL and CY conceived and designed the study and wrote the main manuscript text. SL, XC, and CY conducted the experiments, interpreted the results. XC and SX do the acquisition, analysis, or interpretation of data, helped to write the manuscript. RD drafted the manuscript and revised the paper critically. SW provided the tested strains and was responsible for the background investigation. ZS was the lead investigators, designed the study, supervised the students, and final proofreading. All authors contributed to the article and approved the submitted version.

## Funding

This work was supported by National key R&D plan “Research on modernization of traditional Chinese medicine” (2018YFC1707100).

## Conflict of interest

SW was employed by the company Guangzhou Hipower Pharmaceutical R&D Co., Ltd.

The remaining authors declare that the research was conducted in the absence of any commercial or financial relationships that could be construed as a potential conflict of interest.

## Publisher’s note

All claims expressed in this article are solely those of the authors and do not necessarily represent those of their affiliated organizations, or those of the publisher, the editors and the reviewers. Any product that may be evaluated in this article, or claim that may be made by its manufacturer, is not guaranteed or endorsed by the publisher.
